# Ten steps to investigate a cellular system with mathematical modeling

**DOI:** 10.1371/journal.pcbi.1008921

**Published:** 2021-05-13

**Authors:** Jasia King, Kerbaï Saïd Eroumé, Roman Truckenmüller, Stefan Giselbrecht, Ann E. Cowan, Leslie Loew, Aurélie Carlier

**Affiliations:** 1 MERLN Institute for Technology-Inspired Regenerative Medicine, Maastricht University, Maastricht, the Netherlands; 2 Richard D. Berlin Center for Cell Analysis and Modeling, University of Connecticut School of Medicine, Farmington, Connecticut, United States of America; SIB Swiss Institute of Bioinformatics, SWITZERLAND

## Abstract

Cellular and intracellular processes are inherently complex due to the large number of components and interactions, which are often nonlinear and occur at different spatiotemporal scales. Because of this complexity, mathematical modeling is increasingly used to simulate such systems and perform experiments in silico, many orders of magnitude faster than real experiments and often at a higher spatiotemporal resolution. In this article, we will focus on the generic modeling process and illustrate it with an example model of membrane lipid turnover.

## Introduction

Mathematical models allow testing hypotheses and performing experiments in silico, many orders of magnitude faster than real experiments. In addition, mathematical methods provide us with excellent tools to deal with the large number of biological interactions and nonlinearities within biological systems. But these advantages of mathematical modeling need to be tempered with a full appreciation of the assumptions underlying their construction. The ultimate utility of a model is that its predictions (i.e., simulations) can guide the design of new experiments to test the underlying assumptions. With this overarching theme in mind, the following 10 steps will focus on common aspects of the mathematical modeling process for cell biological systems, without providing an extensive survey of all possible approaches. For more details on various approaches, we refer the reader to [1–6]. The VCell software, an open-source, freely available computational biology platform [7], will be used to illustrate the 10 steps since it includes both an interface that abstracts biological mechanisms (the BioModel interface) as well as an interface that allows the model to be directly formulated as a mathematical description (the MathModel interface). We stress, however, that the 10 steps are generic and could also be applied to other biological systems using modeling packages and software platforms such as COPASI [8], FreeFEM [9], FEBIO [10], COMSOL Multiphysics [11], MATLAB [12], or Python [13].

### Step 1: Understanding the biology of the cellular and intracellular processes to be modeled and studied

**Fig 1:**
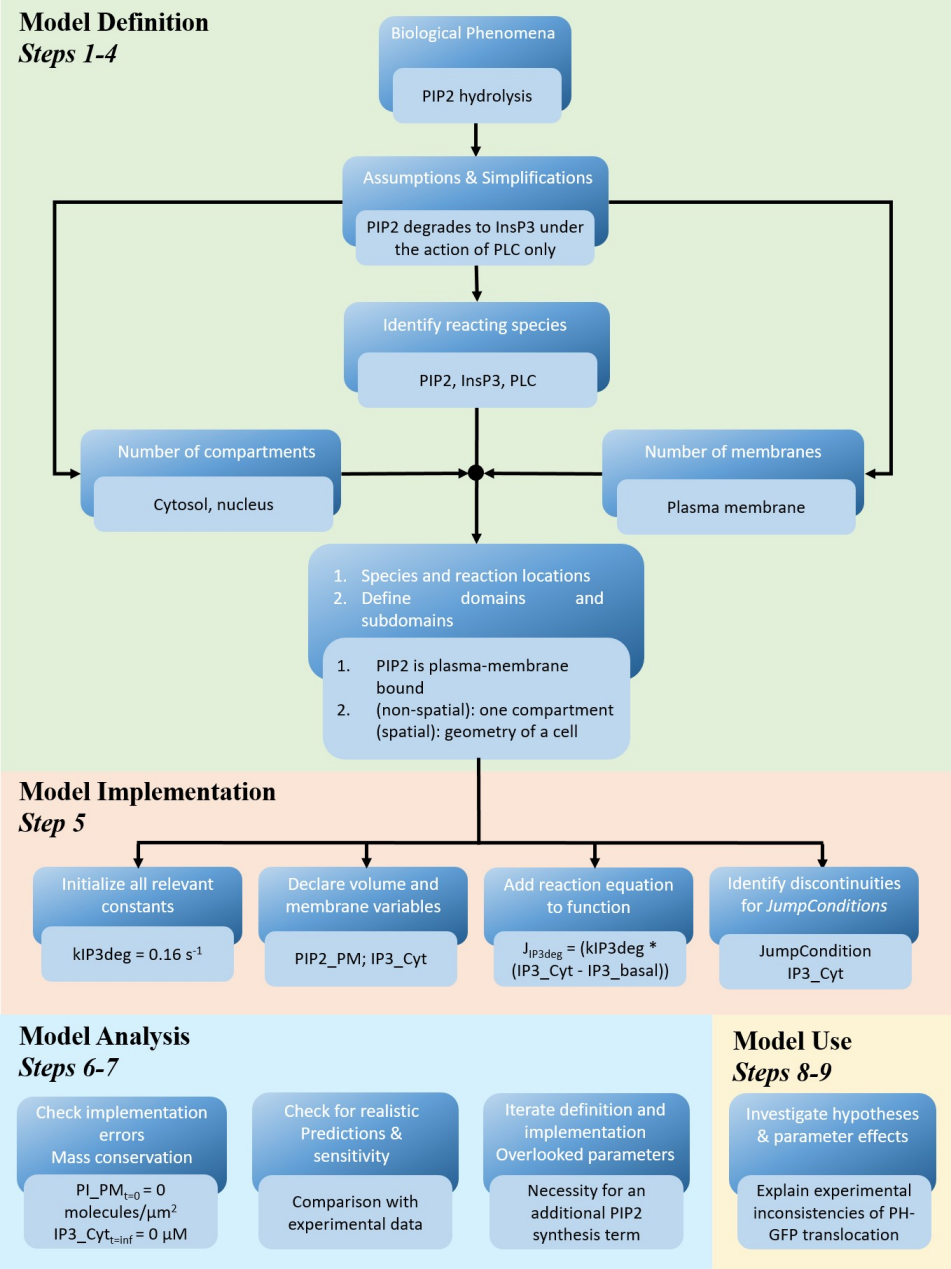
Schematic displaying the workflow for setting up a mathematical model. Before starting the model implementation, modelers must define and simplify the biological phenomena to be simulated. The modeler sets the constants to be called in the equations, defines the membrane and compartment variables, adds needed mathematical expressions as functions, and finally defines all the fluxes at membranes as jump conditions. After the model implementation, the model can be checked for implementation errors and validated, and then used to investigate hypotheses in silico. The dark blue boxes represent the generic workflow, whereas the light blue boxes apply these to the phosphoinositide turnover kinetics example [14].

Although step 1 may sound contradictory to the goal of building a mathematical model (i.e., to enhance biological understanding), a basic, albeit not complete, understanding of the biological system at hand is essential to define and simplify the biological phenomena to be simulated as well as to identify the data available to calibrate and validate the mathematical model. To illustrate the abstract concepts of the 10 steps, we will use phosphoinositide turnover kinetics as an example [14] (see also Fig 1 and S1 Text for the fully worked-out steps). Phosphoinositides, such as PIP2, are membrane phospholipids that play an important role in cell signaling. Experimental evidence showed that PIP2 decreased after bradykinin stimulation and recovered after 2 to 3 min but that the rate of production of its product (InsP3) was much greater than the rate of PIP2 decline. In order to understand this phenomenon, Xu and colleagues [14] first assembled the available data, including the essential (known) components involved in PIP2 hydrolysis and the membrane and cytosolic reactions (see S1 Text for details). They also performed dedicated experiments to determine the relative amounts and basal levels of PI, PIP, and PIP2 as well as the bradykinin-induced changes of PIP2 and PIP in N1E-115 cells as a function of time; the InsP3 production kinetics was known from earlier work [15,16] and was used to further constrain the model.

### Step 2: Simplifying the biology and creating a graphical scheme

*“Everything should be made as simple as possible, but not simpler.”—A. Einstein*

A mathematical model is defined as “an abstract representation of a complex system in terms of equations or rules and a description of the region (spatially and/or temporally) on which they are valid” [17]. Mathematical modeling is the process of creating such an abstract representation and generating novel insights from it. Importantly, the task of creating mathematical models of cell biological systems is not trivial, due to the astonishing complexity and diversity of biological phenomena and the lack of fundamental biological laws. Typically, one will start by summarizing the knowledge of the cell biological system in a graphical manner (see Fig A in S1 Text). In this way, it becomes clear what the key players are (e.g., PI, PIP, PIP2, PLC, DAG, and IP3) and which other players are ignored (e.g., PI 3-kinases, PIP2 5-phophatases). The arrows in the graphical scheme indicate which and how the key players are connected (e.g., the model assumes that PIP2 is produced from PIP only and degrades to InsP3 only under the action of PLC). The graphical scheme can also detail where the key players are located (e.g., PIP2 on the membrane, IP3 in the cytosol). Once the graphical scheme is complete, it can be translated into the mathematical framework.

### Step 3: Selecting the appropriate mathematical model assumptions

In the third step, the graphical scheme is converted into a mathematical model by selecting the proper mathematical model type or approximations (e.g., model scale, static versus dynamic, deterministic versus stochastic, spatially distributed versus spatially homogeneous, or open versus closed) [18]. For example, as the goal of the PIP2 hydrolysis model is to understand the InsP3 production and explain the current mismatch between InsP3 production and PIP2 degradation, a dynamic model, which predicts the concentrations of the variables over time, is required. Depending on the number of interacting molecular species, stochasticity may play a role in the modeled process. However, stochastic models are computationally more expensive and require multiple runs for every in silico experiment, in contrast to a deterministic model where, once all initial conditions are defined, all solutions are fully determined. For the PIP2 model, it was chosen to start simple and simulate the process first deterministically. To capture the overall kinetics, first, a well-mixed compartmental model (ordinary differential equations) was used for the PIP2 model. In a next step, to investigate the spatial variations in PH-GFP translocation after bradykinin-induced stimulation, a spatial model (partial differential equations) was employed. A mathematical model can also be open or closed, depending on whether components can leave or enter the system. This is modeled through the definition of source or sink terms or, in a spatial model, using boundary conditions. For example, to ask whether the PH-GFP translocation is primarily sensitive to PIP2 or to InsP3, the model could simulate how the PH-GFP would respond to only the PIP2 dynamics by keeping the concentration of InsP3 in the cytosol constant (this can be implemented via fast source and sink reactions or, more elegantly, “clamping” a species to a constant or an expression in time). In a spatial model, one can decide whether molecules enter or leave the geometric domain. For example, in the PIP2 model, the molecules do not leave the cell or enter the nucleus, resulting in zero-flux boundary conditions at these boundaries. However, InsP3 in the cytosol is produced by hydrolysis of PIP2 in the plasma membrane, so at this boundary mass conservation is maintained through “jump conditions.” Finally, the scale at which the biological processes occur may also determine the modeling approach. Our example model takes place at the whole cell scale; it does not account for steric effects during bimolecular interactions, which may be ignored for the kinds of questions that are addressed at the whole cell or tissue scale. However, if such details are important, the modeler may need to choose a different approach (e.g., ReaDDY [19] or Spring SaLaD [20]) that takes such details into account at the expense of longer computation times.

### Step 4: Define model components

**Fig 2:**
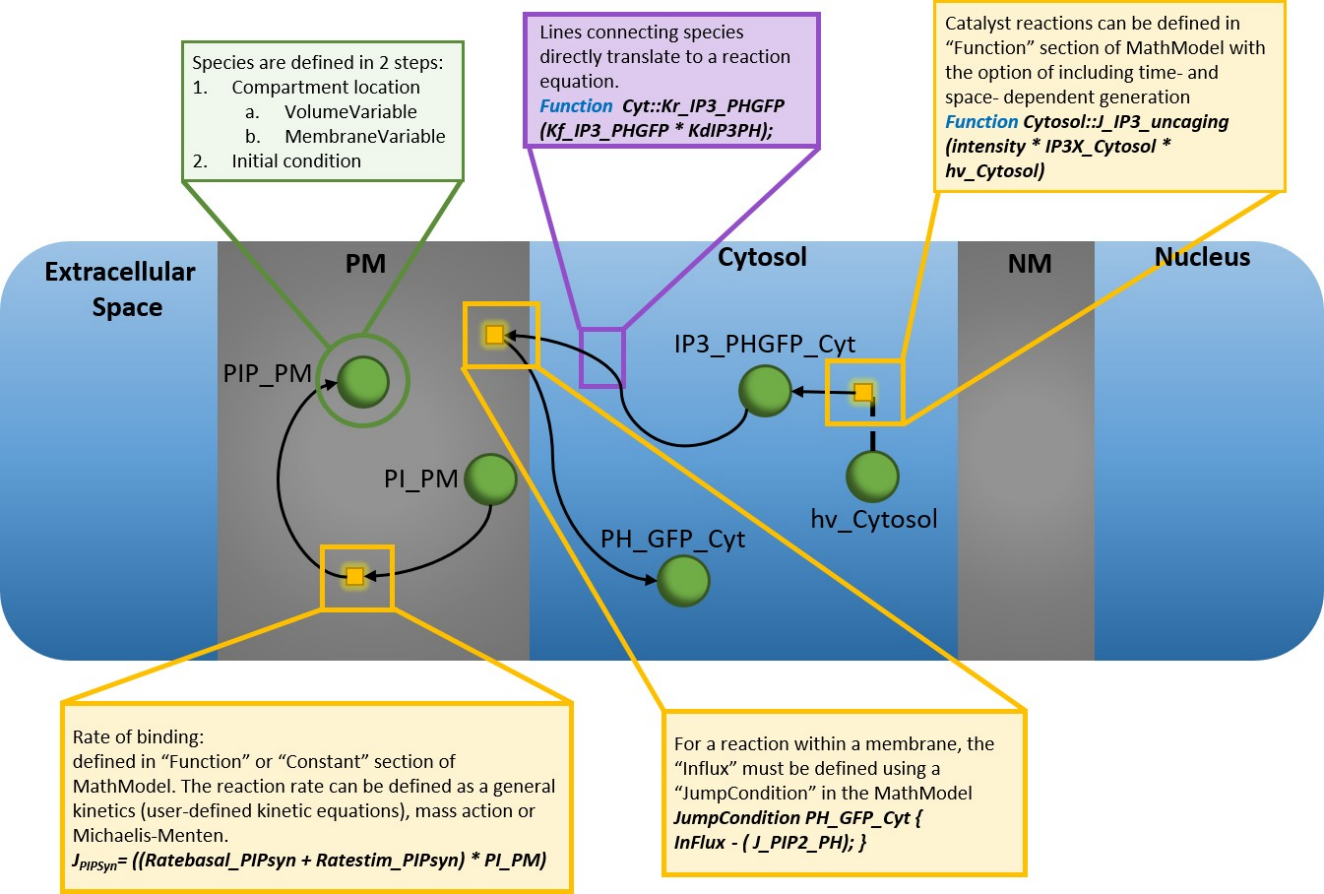
Relationships of the elements of a mathematical model. The schematic visually represents the components of the mathematical model: species (green circle), reaction rates (yellow squares), compartments (Extracellular Space, Cytosol, and Nucleus), and membranes (PM and MM). The arrows connect species together with reaction rates and also define the mass conservation relationships.

Once an appropriate modeling approach has been identified, the model components need to be defined: the variables, parameters, and processes that link the variables (Figs 1 and 2) [2]. The variables represent the biological “species” of interest, such as proteins or metabolites. Species are localized within “compartments” that have defined volume and surface to volume ratios. Processes or “reactions” must then be defined to describe how the variables are linked, such as Michaelis–Menten kinetics to describe the rate of an enzymatic reaction. For example, the PIP2 model has 13 variables (see S1 Text for details), which are localized within the cytosol and plasma membrane, of which the surface to volume ratios have been calculated based on a hemispherical cell of radius 9 μm and a spherical nucleus of radius 3 μm. The PIP2 model also assumes that the reactions occurring between the model variables can be described using mass action kinetics, where the stimulus is modeled with an exponential decay. The parameters within a reaction rate are the fixed numerical constants, such as the binding rate constant for a ligand to a receptor. In this way, the parameters connect the model to reality (see Table A in S1 Text). A variety of methods can be used to determine the parameter values from experimental data for nonspatial models (i.e., inference, optimization, etc. [21]). For example, COPASI (a COmplex PAthway SImulator) [22] has advanced algorithms to optimize parameter values to best fit a set of experimental data. In this optimization task, a given objective function is minimized by scanning one or more parameters over a given value range. COPASI offers many optimization solvers including genetic algorithms, particle swarm, simulated annealing, and steepest descent. In cases where parameter values are experimentally inaccessible or for spatial models where parameter estimation approaches are ad hoc, a sensitivity analysis [23], where multiple simulations are run for different parameter value combinations, can be used to determine the importance of these parameter values on the outcome of interest. If the model is spatial, then the compartmental structure of the problem must be mapped to an appropriate 1, 2, or 3D geometry, and the displacement of the variables (e.g., diffusion) is defined within this geometry (see Fig 2).

### Step 5: Model implementation

Typically, one will first formulate the corresponding set of equations and then convert them into a computer code to solve the set of equations numerically (Fig 1). Many software platforms provide an easy-to-use interface for scientists with limited programing experience to navigate model building; this Graphical User Interface (GUI) can also help to readily communicate the components and structure of the model, once the model is completed and published (see S1 Text for the PIP2 model visualized in the VCell BioModel GUI). Importantly, the user does not need to write lines of code to describe the system of reactions and solve it but only specifies the rate expressions and the parameter values. The software automatically converts the graphical, biological description into a corresponding mathematical system of ordinary and/or partial differential equations on behalf of the user. This automatic conversion significantly reduces the possibility of scripting errors. Experienced modelers may write their own math directly, often with commercial software such as MatLab or Mathematica, but also by coding in languages with large collections of numerics and analysis libraries such as Python. Interestingly, the mathematical representation that is automatically generated from a BioModel application, coded in the VCell Math Description Language (VCMDL), can be viewed, edited, and run independently as a “MathModel.” In the MathModel interface, mathematics-savvy users can directly (1) see the underlying equations, variables, and functions, and (2) edit the corresponding VCMDL code, thereby bypassing the graphical user(BioModel) interface, including, for example, the definition of new variables to store values temporarily or scale them.

### Step 6: Model analysis

Before starting to use the mathematical model, it is important to test the model implementation to: (1) ensure that there are no implementation errors (by checking for example mass conservation); (2) explore the sensitivity of model predictions with respect to the parameter values. For models that explore the response of a system to a stimulus or perturbation, it is important to be sure that all the initial concentrations are at the pre-stimulus steady state (see S1 Text for VCell troubleshooting tips and tricks).

### Step 7: Explore how well the model fits existing experimental results

Comparing the model simulation results to existing (for example, published) experimental results is essential in validating the model. In particular, such a comparison might identify that certain key aspects of the biological process are not captured, requiring a reiteration of steps 2 to 5 to improve the mathematical model. In the case of the PIP2 model, a simple model could not properly reconcile the measured decrease in PIP2 with the previously published increase in InsP3; this necessitated an additional stimulated PIP2 synthesis mechanism to be included in the model (see S1 Text for more information).

### Step 8: Model use

Once the mathematical model has been sufficiently tested and calibrated based on particularly designed experiments, the model can be used to answer “what if?” scenarios, where one might, for example, change the geometry of the compartments, the concentration of the components, their location, or interaction rates. For example, an enzymatic intermediate in a complex pathway can be “clamped” to 0 concentration to perform a virtual “knockout” experiment (see S1 Text for examples from the PIP2 model). One can also perform systematic parameter scans, in which one or more parameters vary according to a predefined list or range of values. This set of in silico experiments can be complemented with an explanation of the changes in parameter values that lead to the observed response. In particular, with an in silico model, the data can be nondestructively recorded at a higher frequency and spatial resolution than in an experimental setting, limited only by a temporal and spatial resolution resulting in a reasonable run time of the numerical simulation.

### Step 9: Test predictions of the mathematical model with experiments

The hypotheses derived from the “what if” simulations can be used to design focused experiments. The simulation results can even suggest experiments that would not have been apparent before the model. For example, the PIP2 model predicted a stimulated synthesis that resulted in an actual increase in PIP2 at an earlier time point than was initially measured and this was subsequently confirmed experimentally [14] (see S1 Text for details). As such, experimentally testable model predictions are the key results of mathematical models, which can lead to new insight into the molecular and biophysical mechanisms underlying the biological process. In turn, the new biological insights lead to new hypotheses that can be explored and analyzed with mathematical models, resulting in an iterative experimental and modeling research cycle.

### Step 10: Share the mathematical model and its implementation

To truly reach the potential of mathematical models and create comprehensive, predictive models of biological systems, models of individual processes will need to be combined. This requires that the individual models are understandable, reproducible, reusable, and composable [24]. EMBL-EBI maintains an extensive repository of models encoded in the SBML standard (https://www.ebi.ac.uk/biomodels/). VCell users can store their models in the VCell database and make them “public,” thereby providing access to the scientific community to the coded form of the mathematical model as well as the settings of the numerical solver to reproduce its analysis. Moreover, other users can save a version of the model, modify it, and combine it with other VCell models. In summary, proper annotation and storage of a mathematical model in a database will promote its reproducibility and future applicability [25–28].

## Conclusions

Mathematical models allow testing hypotheses and performing experiments in silico, many orders of magnitude faster than real experiments. In addition, simulations provide us with excellent tools to deal with the large number of biological interactions and nonlinearities within cellular biological systems. In this article, we highlighted the generic steps of mathematical modeling and how these can be achieved using the VCell package for illustration. The VCell database also enables the reuse and aggregation of mathematical models, which is a promising avenue to reach the full potential of comprehensive, predictive mathematical models of biological systems.

## Supporting information

S1 Text Example model to illustrate the 10 steps. (DOCX)
