# Supplementary material for: Ten steps to investigate a cellular system with mathematical modeling
Source: PLoS Comput Biol. 2021 May 13;17(5):e1008921. doi: 10.1371/journal.pcbi.1008921 (PMC8118325; doi:10.1371/journal.pcbi.1008921)
Supplement: S1 Text — (DOCX) [file pcbi.1008921.s001.docx]

**Supplementary material: Example model**

The example model, used to illustrate the 10 steps, is based on Xu et al., [1].

The Virtual Cell software environment (available at https://vcell.org/) was used to develop a model of PIP2 turnover. The mathematical details of the model are provided in the appendix of the above published paper. The model can be accessed and copied by logging into Virtual Cell and opening the “published” model called “Xu 2003 Kinetic analysis of receptor-activated phosphoinositide turnover” from the VCell database. Opening the model in the VCell software will permit visualization of the time course and/or spatial distribution of all the variables in the model including those that were not presented in this paper. By saving a separate copy, the user can also play with and modify the model.

Tutorials on how to use the VCell software can be found at <https://vcell.org/>.

**Step 1: Understanding the biology of the cellular and intracellular processes to be modeled and studied**

Background knowledge and Experimental data:
See Figure 1 of Xu et al. [1].

- InsP3 increase is a direct consequence of PIP2 hydrolysis induced by PLC activation
- PIP2 decreases after bradykinin-stimulation, and recovers after 2-3 min but the rate of production of its product (InsP3) is much greater than the rate of PIP2 decline
- The cellular level of PIP2 is determined by a balance between degradative and synthetic enzymes
- The majority of PIP2 is synthesized via phosphorylation of PI (to PIP to PIP2)
- The degradation of PIP2 can be mediated by PLC, PI 3-kinases, or PIP2 5-phophatases such as synaptojanin.
- The recovery of membrane PIP2 is hypothesized to be mediated by PIP2 resynthesis at the plasma membrane.
- A PH-GFP, a probe for PIP2, has a high affinity for PIP2 and InsP3. The probe predominantly associates with the plasma membrane in unstimulated cells and translocates to the cytosol on activation PLC. Since the PH-GFP probe shows a 10–20-fold higher affinity for InsP3 than PIP2 in vitro, it is not clear whether the translocation of PH-GFP from membrane to cytosol is due to a decrease in membrane PIP2 level or an increase in cytosolic InsP3.

Open questions:

- How can there be much more InsP3 production than PIP2 decline?
- Is there resynthesis of PIP2 at the membrane? By what is this mediated?
- What determines the translocation of PH-GFP from the membrane to the cytosol?

**Step 2: Simplifying the biology and creating a graphical scheme**

Figure A provides a graphical overview of the PIP2 model. The key players are called “species” in VCell and in the BioModel, the graphical user interface environment of VCell, they are represented by a green circle (see Fig A). It is clear that for the particular research questions some other players are ignored (e.g. PI 3-kinases, PIP2 5-phophatases). “Species” are localized within “compartments” that have defined volume and surface areas (e.g. cytosol and PM in fig A). The processes, called “reactions” in VCell (yellow squares in Fig A), describe how the variables are linked. The variables involved in the reaction are linked to or from the reaction with arrows indicating the forward direction for the reaction rate (see Fig A). Note that in the proposed PIP2 model it is assumed that PIP2 is produced from PIP only, and degrades to InsP3 only under the action of PLC. The model also assumes (see also steps below) that the PH-GFP-bound forms of InsP3 and PIP2 do not degrade.


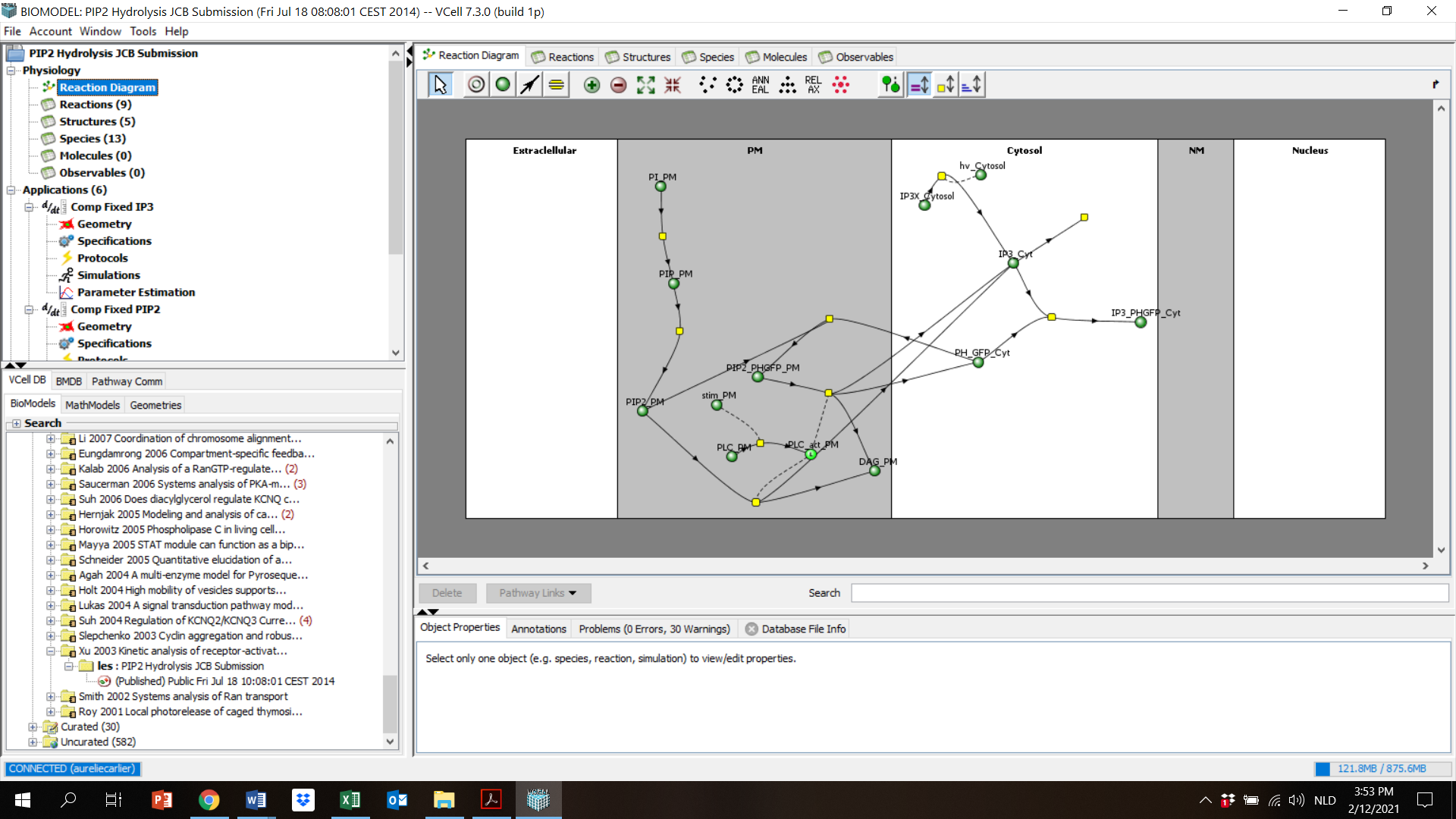


Figure A: Biomodel Reaction diagram of the PIP2 model.

**Step 3: Selecting the proper mathematical model type**

Selections for the PIP2-model:
- deterministic: this assumes that the concentrations of the variables are high enough so that a continuum approximation is valid

- non-spatial (ordinary differential equations) & spatial (realistic cell geometry, partial differential equations)
- scale: intracellular scale; the variables are modeled as continuous concentrations in the cytosol volume and surface densities on the membrane

- dynamic: the variables change over time, this is important to be able to capture the peak concentrations and timing thereof

- open: some species may be clamped, meaning that there concentrations are kept constant by allowing production or degradation

**Step 4: Define model components**

Variables:

PH_GFP_cyt
IP3_PHGFP_cyt
hv_cytosol
IP3X_cytosol
IP3_cyt
PIP2_PHGFP_PM
PI_PM

Stim_PM
PIP2_PM

PIP_PM

DAG_PM

PLC_PM

PLC_act_PM

Parameters:

Table A provides some examples of parameter values used in the PIP2 model, for a full list of parameters, see Xu et al. [1]. Note that some values are measured, others are calculated based on geometrical approximations, taken from literature or fitted to the experimental data.

Table A: PIP2 model parameters (adjusted from Xu et al. [1])

| **Parameter** | **Value** | **Comments** |
| --- | --- | --- |
| PIP2_basal | 4000 molecules/µm^2^ | measured |
| PIP2syndecay | 2857 molecules/µm^2^ | Best fit to fig 1 in Xu et al. [1], appropriate for the required rate of InsP3 production |
| SurfToVol_NM | 1 | Spherical nucleus of radius 3 µm |
| SurftoVol_PM | 0.5 | Calculated for a hemispherical cell of radius 9 m; the N1E-115 neuroblastoma cell are often significantly larger but with their many processes and convoluted membranes this makes a good estimate |
| kBasalSynPIP2 | 0.048 s^-1^ | Chosen to produce recovery phase of PIP2 (Fig 1, Xu et al. [1]) |
| kStimSynPIP2 | 0.92 s^-1^ | Fit to experimental data in Fig 1 |
| k_PIP2hyd | 2.4 (s.molecules/µm^2^)^-1^ | Appropriate for required rate of InsP3 production |
| tauPIP2syn | 0.05 s | Stimulation introduced at 50ms |
| kdPIP2PH | 2 µM | Hirose et al. [2] |
| Kf_PIP2PH | 0.12 (µM.s)^-1^ | Van der Wal et al. [3] |

Processes:
We will focus here on some examples to illustrate the general structure of the equations. The entire equation system can be found in Xu et al. [1]. Note that plasma membrane species are designated by PM suffixes, and cytosolic species with Cyt suffixes. Typically, one will define one equation per variable, and that equation will have as many terms as the number of ways in which that variable can change its concentration. For example, the concentration of PIP2_PM can change due to PIP2 hydrolysis, PH-GFP binding, PIP2_PHGFP complex dissociation and PIP2 synthesis, resulting in four terms in equation S1. If a particular process increases the concentration (e.g. PIP2 synthesis), the term will have a positive sign. Similarly, if a particular process reduces the concentration (e.g. PH-GFP binding), the term will have a negative sign.

*Non-spatial deterministic model: ordinary differential equations:*

$$\frac{{dPIP2}_{PM}}{dt}=\underset{PIP2 hydrolysis}{\underbrace{-k_{PIP2hyd}.PIP2_{PM}.PLC_{act_{PM}}}-}\underset{PH_{GFP}binding}{\underbrace{k_{fPIP2PH}.PIP2_{PM}.PH_{GFP_{Cyt}}}+}$$

$$\underset{PIP2-PHGFP dissociation}{\underbrace{k_{rPIP2PH}PIP2_{PHGFP_{PM}}}+}\underset{PIP2 synthesis}{\underbrace{\left( PIP2_{synbasal}+{PIP2}_{synstim} \right).PIP_{PM}}}$$

(Eq. S1)

$PIP2synbasal=(0.581.\left( PIP2_{PM}<PIP2_{basal} \right).k_{basalsynPIP2}.(-1+e^{\frac{\left( PIP_{basal}-PIP2_{PM} \right)}{PIPbasal}})$

(Eq. S2)

$$PIP2synstim=k_{stimsynPIP2}.e^{\frac{-\left( t-tauPIP2syn \right)}{PIP2syndecay}.(t>tauPIP2syn)}$$

(Eq. S3)

Note that the PIP2 hydrolysis and PH_GFP binding and unbinding is modeled with mass action (Eq. S1). If the PIP2_PM concentration is lower than a PIP2basal-level (this is a pre-determined parameter setting), there is basal synthesis rate (Eq. S2). After a tauPIP2syn timed delay (also a pre-determined parameter setting), there is also stimulated synthesis of PIP2 (Eq. S3).

*Spatial deterministic model: partial differential equations:*

$$\frac{{\partial PIP2}_{PM}}{\partial t}=PIP2 diffusion+PIP2 reaction$$

$$=\underset{PIP2diffusion}{\underbrace{PIP2_{Diff}.\nabla^{2}(PIP2_{PM})}}\underset{PIP2 hydrolysis}{\underbrace{-k_{PIP2hyd}.PIP2_{PM}.PLC\_act\_PM}-}\underset{PH_{GFP}binding}{\underbrace{k_{fPIP2PH}.PIP2_{PM}.PH_{GFP_{Cyt}}}}$$

+ $\underset{PIP2-PHGFP dissociation}{\underbrace{k_{rPIP2PH}PIP2_{PHGFP_{PM}}}+}\underset{PIP2 synthesis}{\underbrace{\left( PIP2_{synbasal}+{PIP2}_{synstim} \right).PIP_{PM}}}$ (Eq. 4)

For compartmental (i.e., nonspatial) models, diffusion is considered to be instantaneous on the time scale of interest, and the equations reduce to a set of ordinary differential equations containing just the reaction rates with appropriate adjustments for the surface-to-volume ratio of the plasma membrane to the cytosol (SurfToVol_PM). For a spatial model, the diffusion is explicitly captured and a diffusion coefficient needs to be set for all diffusible species.

**Step 5: Model implementation**

VCell Biomodel interface

The BioModel provides a graphical user interface environment where the following model elements can be defined: “physiology”, “pathway”, “applications” and “simulations”. “Physiology” is a conceptual (graphical) representation of the biological system in terms of cellular structures (e.g. cytosol, nucleus, or plasma membrane), species and biochemical reactions occurring within or outside the cell. The user can build the model reactions by dragging and dropping reacting species to their reacting compartments. Arrows representing the forward direction of the reactions taking place connect these species accordingly (Fig A).

Note that in a VCell spatial model, volume “compartments” must always be separated by “membranes”. “Applications” define the quantitative conditions needed to simulate a virtual experiment such as the initial conditions, diffusion coefficients, geometries (i.e. well-mixed or spatial, 1D, 2D or 3D), numerical solver (stochastic, deterministic, hybrid, network-free), etc. A single “BioModel” may have numerous “Applications”, in which geometries, initial conditions or mathematical methods have been changed. Each “Application”, in turn can have multiple “Simulations”, in which different simulation parameters are used.

VCell MathModel Interface

To create a MathModel, the user can start *de novo* (File > New > MathModel), or from a BioModel Application and convert it into a MathModel. To convert the PIP2 model to a MathModel, select a particular application > Simulations > Generated Math > Create MathModel. This conversion creates a general framework and allows understanding the general VCMDL syntax. Within the VCMDL Editor, the user can initialize the constants to be called in the equations, define the membrane and compartment variables, add the equations in the function section, and finally define all the discontinuities as jump conditions. If an initial condition or parameter is not constant, the “function” feature can be used. VCell also specifies a set of reserved symbols and constants that can be used in expressions and computations. Note that VCell will entirely convert the BioModel implementation.

MathModel implementation:
Compartmental PIP2

Function Compartment::J_PIP2_PH ((PIP2_PM * kf_PIP2PH * PH_GFP_Cyt) - (kr_PIP2PH * PIP2_PHGFP_PM));

Function Compartment::Rate_PIP2_hyd (k_PIP2hyd * PIP2_PM * PLC_act_PM);

Function Compartment::Rate_PIP2Syn ((Rate_PIP2Synbasal + Rate_PIP2SynStim) * PIP_PM);

Function Compartment::Rate_PIP2Synbasal (0.581 * (PIP2_PM < PIP2_basal) * kBasalSynPIP2 * ( - 1.0 + exp(((PIP2_basal - PIP2_PM) / PIP2_basal))));

Function Compartment::Rate_PIP2SynStim (kStimSynPIP2 * exp( - ((t - tauPIP2syn) / PIP2syndecay)) * (t > tauPIP2syn));

CompartmentSubDomain Compartment {

OdeEquation PIP2_PM {

Rate ( - Rate_PIP2_hyd - J_PIP2_PH + Rate_PIP2Syn);

Initial PIP2_PM_init_molecules_um_2;

}
}

Spatial PIP2

Function Cyt_EC_membrane::Rate_PIP2_hyd (k_PIP2hyd * PIP2_PM * PLC_act_PM);

Function Cyt_EC_membrane::Rate_PIP2Syn ((Rate_PIP2Synbasal + Rate_PIP2SynStim) * PIP_PM);

Function Cyt_EC_membrane::Rate_PIP2Synbasal (0.581 * (PIP2_PM < PIP2_basal) * kBasalSynPIP2 * ( - 1.0 + exp(((PIP2_basal - PIP2_PM) / PIP2_basal))));

Function Cyt_EC_membrane::Rate_PIP2SynStim (kStimSynPIP2 * exp( - ((t - tauPIP2syn) / PIP2syndecay)) * (t > tauPIP2syn));

MembraneSubDomain Cyt EC {

Name Cyt_EC_membrane

BoundaryXm Value

BoundaryXp Value

BoundaryYm Value

BoundaryYp Value

OdeEquation PIP2_PM {

Rate ( - Rate_PIP2_hyd - J_PIP2_PH + Rate_PIP2Syn);

Initial PIP2_PM_init_molecules_um_2;

}

Time delay and multiple time scales

Note that neither VCell nor COPASI explicitly handle time delays, but both software will correctly solve the underlying detailed biochemistry that produces an apparent time delay. Multiple timescales are generally handled by “stiff” solvers for both ODE and PDE simulations that use adaptive time steps; in particular, the default ODE and PDE solvers in VCell are stiff solvers with variable time steps. VCell also offers a choice of fixed timestep solvers that can be used for multiple time scales by identifying which part of the system is sufficiently fast so that it can be solved algebraically (pseudo-steady approximation).

**Step 6: Model analysis**

VCell checks for internal consistency and may highlight errors and warnings. Errors must be corrected before a MathModel can be saved to the database and run. Warnings indicate potential problems that VCell has identified in the model, but do not prevent the user from saving the file, generating the math and running the simulations. To further troubleshoot, it might be interesting to compare previous versions of the code. This can be facilitated by copy-pasting the generated math description into word-processor files (.doc or .txt) so that an automatic comparison tool can be used to detect differences. Alternatively, VCell also provides a tool for comparing VCell models (File > Compare with Saved). Additional information can also be found in the “Help” menu bar option, which links to the VCell website, the VCell Open Discussion forum and VCell Support contact details. To help troubleshooting, it is possible to grant access to specific users (File > Permissions). To assure that the system is at steady state, an Application can be created where the stimulus is turned off; if the simulation shows that the variables change over time, the system was indeed not at steady state. The modeler can let these simulations run until all variables remain stable and then copy these steady values into the initial specifications in an Application where the stimulus is applied.

**Step 7: Explore how well the model fits existing experimental results**In case of the PIP2 model, a simple model consisting of transient activation of PLC-mediated PIP2 hydrolysis followed by a slow recovery to basal levels via phosphorylation of PI and PIP, could not account for sufficient InsP3 unless a stimulated synthesis of PIP2 was added (see Eq. S3 above). The new model subsequently predicted a transient increase of PIP2 which was confirmed by additional experimental measurements at earlier time points than in the initial data set (see Figure 1 Xu et al. [1]).

**Step 8: Model use**

Once the compartmental model was calibrated with experimental data, it was extended to include PH-GFP binding (see figure 5, Xu et al. [1]). The model was also made spatial in order to be able to compare the PH-GFP translocation with imaging data (Figure 6-7, Xu et al. [1]). Interestingly, the spatial simulations indicate that the nucleus creates a small diffusion barrier, resulting in a high surface-to-volume region and a temporary buildup of released PH-GFP in the upper right corner of the cell (observed both experimentally and in the simulation).

Note that VCell has a convenient “parameter scan” option in the “Simulations” that allows running simulations in which one or more parameters vary according to a predefined list or range of values. In the “Simulations” tab, the spatial resolution (mesh size) as well as temporal resolution (step size, and saved time points) can be adjusted.

**Step 9: Test predictions of the mathematical model with experiments**

Xu et al. [1] used the PIP2 model to examine the contradicting experimental results that were reported when InsP3 was introduced into cells in the absence of receptor-mediated activation of PLC. More specifically, Hirose et al. [2] reported that injection of 1 µM InsP3 could produce large translocation of PH-GFP, whereas van der Wal et al. [3] reached the opposite conclusion when using photorelease of caged InsP3. More specifically, by simulating the translocation response for different concentrations of cytosolic PH-GFP (see figure 9 Xu et al. [1]), Xu et al. found that the translocation effect is very sensitive to the expression level of the indicator PH-GFP. As such, the inconsistency in the experiments of Hirose et al. [2] and van der Wal et al. [3] may be explained by differences in indicator concentrations.

**Step 10: Share the mathematical model and its implementation**

The Virtual Cell software environment (available at https://vcell.org/) was used to develop a model of PIP2 turnover. The model can be accessed and copied by logging into Virtual Cell and opening the “published” model called “Xu 2003 Kinetic analysis of receptor-activated phosphoinositide turnover”. Opening the model in the VCell software will permit visualization of the time course and/or spatial distribution of all the variables in the model including those that were not presented in this paper. By saving a separate copy, the user can also run and modify the model.

Note that VCell users can store their models in the VCell database and make them public. Users can load an entire model into their own workspaces or just copy and paste individual reactions. All components of the VCell Physiology (species, compartments, reactions) can be annotated to properly identify the components. Within the VCell MathModel, it is possible to use /** **/ or /* */ to comment the code and make it understandable.

**Bibliography**

1. Xu C, Watras J, Loew LM. Kinetic analysis of receptor-activated phosphoinositide turnover. J Cell Biol. 2003;161: 779–791. doi:10.1083/jcb.200301070

2. Hirose K, Kadowaki S, Tanabe M, Takeshima H, Iino M. Spatiotemporal dynamics of inositol 1,4,5-trisphosphate that underlies complex Ca2+ mobilization patterns. Science (80- ). 1999;284: 1527–1530. doi:10.1126/science.284.5419.1527

3. Van der Wal J, Habets R, Várnai P, Balla T, Jalink K. Monitoring Agonist-induced Phospholipase C Activation in Live Cells by Fluorescence Resonance Energy Transfer. J Biol Chem. 2001;276: 15337–15344. doi:10.1074/jbc.M007194200
